# Caring for Frail Older People Living Alone in Italy: Future Housing Solutions and Responsibilities of Family and Public Services, a Qualitative Study

**DOI:** 10.3390/ijerph19127413

**Published:** 2022-06-16

**Authors:** Maria Gabriella Melchiorre, Barbara D’Amen, Sabrina Quattrini, Giovanni Lamura, Marco Socci

**Affiliations:** Centre for Socio-Economic Research on Ageing, IRCCS INRCA—National Institute of Health and Science on Ageing, 60124 Ancona, Italy; g.melchiorre@inrca.it (M.G.M.); s.quattrini@inrca.it (S.Q.); g.lamura@inrca.it (G.L.); m.socci@inrca.it (M.S.)

**Keywords:** ageing in place, frail older people, living alone, housing solutions, home, nursing home, caring responsibility, family, public services, Italy

## Abstract

When frail older people age alone in place, with increasing functional limitations, they require support in performing daily living activities. In this respect, it is important to assess their preferences in terms of future housing solutions, and their opinions/orientations on the care responsibilities of both family and public services. The present study aimed to explore these aspects in Italy. Qualitative interviews were carried out in 2019 within the “Inclusive ageing in place” (IN-AGE) research project, involving 120 frail older people who lived at home in three Italian regions (Lombardy, Marche, and Calabria). A content analysis was conducted, and some quantifications of interviewees’ statements were provided. The results revealed that the majority of seniors prefer ageing at home, at least with a personal care assistant (PCA), whereas moving to a nursing home is typically deemed as a last option. Moreover, they considered the family to be primarily responsible for taking care of them, even with the support of public services. In addition, some territorial differences emerged. Strengthening an integrated model of long-term care (LTC) for older people, where both formal and informal supports allow frail older people to age at home, seems thus a good overall policy solution to pursue, with interventions based also on the needs and preferences of both seniors and their respective families.

## 1. Introduction

For older people living alone, supporting their ability to age in place, e.g., in their own home [1], becomes crucial when limitations in their physical functionalities compromise performing the activities of daily living. Thus, community-dwelling older adults, especially those with limited informal and formal care supports, face a high risk of becoming frail [2], and during the experience of transitioning toward residential care facilities, older people face substantial challenges (e.g., changes life patterns, isolation, loss of autonomy, stress) [3].

Frailty is “an ageing-related syndrome of physiological decline, multisystem dysfunction, and susceptibility to adverse consequences” [4] (p. 2), representing a crucial public health challenge affecting population ageing [5,6], being a geriatric syndrome that increases vulnerability to adverse health outcomes [7,8]. The interaction between older age and chronic diseases can result in increased frailty, which in turn can lead to disability, hospitalization, and also death [9,10,11]. The literature often relates the physical characteristics of frailty to difficulties in performing basic and instrumental Activities of Daily Living (ADLs and IADLs) [12,13]. As people age, the level of independence in performing ADLs e IADLs indeed decreases [14]. The higher level of functional limitations in people aged 80 and over has also been noted [14,15]. Several instruments/tools for measuring frailty have been developed, in order to provide a clinical assessment in different settings [7], but most of them are appropriate for detecting general health outcomes, and not all dimensions of frailty [16]. The results of a systematic review and meta-analysis, reporting a pooled prevalence of frailty among older people near to 18% in all settings, showed that frailty is a common issue in European countries, even though differences emerged according to settings and definitions of frailty itself [17]. A systematic review on frailty in nursing homes [18] indeed estimated a prevalence in the range of 19–76%, whereas other authors [19] found a prevalence between 4–59% in community-based studies, with a total weighted prevalence of approximately 11% in people older than 65 years. In particular, in high-income countries, frailty is estimated to affect 10% of community-dwellers aged 65 years and over, and 25–50% of those aged 85 and over [6,19]. Previous literature [20] found the lowest levels/prevalence of frailty in the population over 65 years in Austria and Sweden (11% and 9%), and the highest in Spain and Italy (27% and 23%).

In the latter country, where this study was carried out, the proportion of over 65 s was about 24% of the total population as of 1 January 2021 (i.e., the highest value among European countries), with 50% of people living alone aged over 65 years, and 44% of this population group having severe functional limitations and great difficulties in performing ADLs [21]. In such a context, belonging to a family-based care regime, the long-term care (LTC) system provides services such as home care, residential care, and especially cash benefits. However, in Italy, frail older people receive support mainly from relatives, in particular children/daughters [22,23]. Public services remain indeed overall marginal, with only 1% of older people benefiting from home care service (SAD, *Servizio di Assistenza Domiciliare*), and only 2% staying in residential care facilities, in 2018. In the same year, conversely, the national disability attendance allowance (IA, *Indennità di accompagnamento*), accounted for 12% of older users [24]. In the European Union (EU 27), the average shares of older people aged 65 years and over receiving institutional care, home care, and cash benefits, were, respectively about 4%, 6%, and 9% [25]. In Italy, territorial/regional differences also emerged in public care for older people, with overall prevalence of IA in the South, IA and SAD in the Centre, and residential care in the North [26]. However, as for daily home care for seniors aged 75 years and over living alone, the situation is partly different, with 1.5% of users in the North, 0.3% in the Centre, and 0.7% in the South [27]. The overall scarce/lacking public care support in Italy, especially concerning home care, is replaced by personal care assistants (PCAs), 44% of whom are from Eastern Europe, and often remunerated (also) with the IA (when available), and hired with verbal agreement/without a regular work contract [28]. There were a total of 407,000 regular PCAs (i.e., having a formal work contract) in 2019 and about 40% of them were concentrated in three regions (Lombardy and Tuscany in the North, Emilia-Romagna in the Centre) [29].

Moving on from available supports to seniors’ opinions for housing solutions/preferences, the literature provides evidence that staying at home and ageing in place, with help from family or public services, is preferred to moving to a care/nursing home. This is considered as the last/not wanted option, because it implies the loss of both autonomy and independence [30,31,32,33]. Other authors suggest that retirees expect to age in place, especially after living in their home for several years [34]. Nursing homes were preferred by male older people with low health status, whereas women preferred relatives’ homes [30]. More recent findings [35,36,37] highlight how the decision-making process, regarding moving from home to an alternative housing arrangement, is difficult and complex, with a great diversity of possible preferences. The decision to remain/stay at home or not, and the associated factors leading to the institutionalization of older people, are influenced by chronic health conditions, the physical functionalities level, care/health needs, availability of support from relatives, the context of welfare resources, and attachment to own place. These recent findings confirmed similar previous results regarding the links between poor health and moving to a different environment [38,39], and attachment to one’s home/neighbourhood and ageing in place, thus implying affective aspects [1], the importance of memories [40], and desires of independence [41]. Moreover, the low availability of social support networks negatively impacts the decision of older people to age in place, especially of older widowers living alone [42]. This highlights that older persons in Northern and Western Europe more often choose to live in residential facilities, as compared to Southern European countries such as Italy, where the family is a pillar of caregiving [43]. In this country, a strong attachment to one’s own home as a place for ageing also emerged [44].

In relation to seniors’ opinions on caring responsibilities of both family and public services, the cultural assumption that children will take care of their older parents is high in Latin societies, such as Italy, and it seems more a social expectation than a voluntary decision of children themselves [45]. There is a reliance on traditional family support, since family/children caregiving is considered as a “moral obligation”, particularly a duty of female members, without relying on private/paid help for providing domestic work [36], even though social, economic and value changes in recent years are eroding the traditional key care role of families. However, there are also seniors who conversely do not consider caring as a family duty and relatives as the elected providers of support, especially in Northern Europe, where welfare systems work well and seniors receive sufficient support from public services, in addition to help from friends and neighbours [36]. Thus, some seniors consider their home as a “private space”, where traditional caregiving can only be provided by the family, whereas for others it is an “open space”, where public and private support can be combined. Additionally, professionals highlighted that caring responsibility should ideally be transferred from the family to the community, in order to permit older people to remain at home [36,46].

In order to explore the opinions of frail older people with physical limitations living alone in Italy related to future housing preferences/solutions, regulatory public/private orientation on care, and current/received care arrangements, this paper aimed to answer the following research questions: (1) What are seniors’ preferred future housing solutions (e.g., ageing in place at home, at home with PCA, in nursing home)? (2) Should caring responsibilities be the main duty of the family and/or of public services? (3) Are there links between these opinions and socio-demographic characteristics of respondents (e.g., gender, living situation, mobility and supports received)? (4) Are there regional differences in this respect? The analysis of these opinions can be of help in order to understand possible links among available care, preferred care, and responsibility of care for frail older people in Italy, with practicable insights for policy makers.

## 2. Materials and Methods

### 2.1. Study Design and Participants

The results presented come from the “Inclusive Ageing in Place” (IN-AGE) study, whose design is based on the Fundamental Qualitative Description, and involved 120 Italian older people (both men and women) aged 65 years and over. They were recruited from three Italian regions: Lombardy (North), Marche (Centre) and Calabria (South/Midday). These regions were chosen as representative of different levels of socio-economic development of this country [47], respectively, of high, medium and low socio-economic development, following the “*Three Italies*” scheme of Bagnasco [48], recently confirmed also by the OECD [49]. In each region, one medium-sized urban city (Brescia, 197,000 residents; Ancona, 100,000; and Reggio Calabria, 172,000, respectively) [50], and one inner/rural area [51] (Oltrepò Pavese, Appennino Basso Pesarese e Anconetano, and Area Grecanica, respectively) were included. Moreover, the most fragile locations within both urban and rural contexts were detected, according to the following criteria: greater presence of older people living alone and families living in public housing (*Edilizia Residenziale Pubblica*—ERP), in addition to a high unemployment level, low education level, and low provision of public services, especially for older people [52]. Drawing on the literature reported above, our study analysed frailty as a condition linked to ageing with limited functional abilities, and a consequent reduction in independence and increased need for support, especially when living alone [53]. The specific inclusion criteria for participants recruited in the study were as follows: older people living at home alone or with the support of a PCA; mobility within the home and outside with help (from a person or aids); no cognitive impairment, in order to answer interview questions independently; and absence of very close family members (living in the same urban block/rural building) providing support for daily activities. Following these criteria, a purposive sample [54] of 72 respondents from urban cities (24 each), and 48 from rural sites (16 each) was interviewed (40 in each region). The recruitment of respondents was carried out with the help of local sections of voluntary associations (e.g., Auser, Anteas, Caritas), and operators of SAD. These recruiting channels informed potential eligible participants on the purpose of the study, collected preliminary adhesions, and obtained the verbal consent of seniors to communicate their personal contact details (address and telephone numbers) to interviewers.

### 2.2. Data Collection and Measures

Qualitative face-to-face interviews were administered in May-December 2019 by six researchers (two for each region, mainly psychologists and sociologists), using a semi-structured interview/topic guide, with questions on general socio-demographic aspects, health status and functional limitations, care arrangements and use of services, housing and the economic situation of respondents. The topic guide was based on questions adapted from previous similar studies, e.g., [55], in addition to validated scales to detect functional limitations, i.e., ADLs and IADLs scales [56], and questions on both sensory (difficulty in seeing and hearing) and mobility limitations (going up/down the stairs, and bending to pick up an object) [57,58]. As for preferred future housing solutions, the questions were as follows: (1) What do you think about the possibility of ageing in place, in particular if health problems should emerge/worsen? (2) Would you prefer to age in your home? (3) If not, what might be the best future housing solution for you (e.g., nursing home)? For opinions on caring responsibilities, the questions were as follows: (1) With regard to the difficulties that older people may encounter in daily life, do you think that supporting them should be a main task of families/children? (2) Should help from public services be exclusive or greater, in order not to ask too much help from family/children?

### 2.3. Ethical Considerations

The approval of the Ethics Committee of Polytechnic of Milan was obtained (POLIMI, Research Service, Educational Innovation Support Services Area, authorization n. 5/2019, 14 March 2019), and written informed consent was signed by each participant before being interviewed. Participants were reassured on the confidentiality/privacy and anonymity of the information collected, according to ethical issues indicated by the European General Data Protection Regulation (GDPR) n. 679, of 27 April 2016 [59].

### 2.4. Data Analysis

Narratives were audio-recorded and transcribed verbatim by interviewers. The identity of respondents, i.e., name, address and telephone number, as direct potential identifiers, was replaced with alphanumeric codes [22]. Then, as steps of the Framework Analysis Technique [60,61], the following activities were performed: reading the transcribed narratives; identification of macro-sub categories/themes; indexing-labelling; construction of a thematic chart; and interpretation of contents. A thematic content analysis was carried out [62]. This phase was performed manually, without the support of any software, as also indicated in the literature [63,64], and was facilitated by the use of questions included in the interview as a preliminary conceptual framework/guide, since they were drawn from theoretical-based categories relevant to the topic of the study, and derived from the literature and experience of researchers [22,65]. Additionally, the cell color-coded process was applied, in order to group data based on the color assigned to each category/theme [66]. Two members from each research team (respectively from Lombardy, Marche and Calabria) filled in the charts with sections from 40 interviews of the respective region (then merged), and analysed the results of all 120 narratives in three regions, with regard to themes closest to their expertise. All researcher of the project’s consortium discussed the appropriateness of reading/coding the contents [22]. Moreover, qualitative dimensions were quantified for better presenting the results, as a “complement to an overall process orientation to the research” [67] (p. 6), and were elaborated (mainly bivariate analyses) using Microsoft Excel 2019 (Microsoft Corporation, Washington, DC, USA), in order to explore, more in depth, any possible relations (e.g., with gender, mobility, level of functional limitations). Some findings are also presented in Section 3 as absolute values/simple frequency of labels in selected answers (without a reference table), when referred to by a few participants (e.g., opinions on efficiency of public service). For the analysis, the following main categories/labels were examined and also quantified (Table 1).

Thus, the overall analysis combines/alternates quantitative and qualitative findings, with some regional differences, when relevant. Original statements/verbatim quotations are also included [68,69], and coded with the first three initials and the progressive interview number (1–40) of the respective region (LOM = Lombardy; MAR = Marche; CAL = Calabria). Irrelevant omissions were placed in round brackets, and some words/terms were added, as represented in the square brackets, in order to help understand the quotations. Further details on the Methods (setting, sampling, participants, data collection, measures, and data analysis) can be found in a previous publication [22], from which Section 2 has been partly adapted.

## 3. Results

### 3.1. Sample Characteristics

The sample included 120 older participants, who were mainly aged 85 years and over, women, with an elementary level of education, and widowers. Moreover, most participants lived alone, and with a PCA only in 27 cases. Regarding functional status, a greater mobility outside the home with help, and both a mild and very high level of physical limitations emerged. Help for providing the activities of daily living largely derives from the family, especially from children, and less from public services, mainly SAD. At the regional level, in Lombardy, more respondents who were able to go outside their home and living alone were identified, whereas in Calabria a greater number of older people aged 85 years and over, with an elementary level of education, living with a PCA, and with a serious level of physical limitations emerged. Additionally, in this region, more so than in both Lombardy and Marche, families were often found to support relatives needing care, while little help from public services (Table 2).

The monthly income is concentrated in the bracket 600–1500 EUR, without particular differences among the three regions. On the whole, the sample shows some opposite aspects between North and South, whereas the Centre of Italy, Marche in this case, is an intermediate context. More details on the sample are available in a previous publication [22].

### 3.2. Future Housing Solutions for Older People

#### 3.2.1. Different Possibilities

A general request to express oneself in terms of preferred future assistance in older age was formulated, specifically if health problems should emerge/worsen (Table 3).

The most accredited hypothesis, as a preferred choice, is being able to stay/remain at home (51%), also with the support of a PCA (20%). No one indicated the preference to stay at home with help from public care services, e.g., SAD. It should be noted that among those who express the second option, only 6% already had a PCA, thus mainly those without PCA (14%) underline the importance of ageing in place, with constant help from a private assistant as a needed resource. A minority of 30% also indicated future transferral to a nursing home, but of these just 7% considered it as first choice, and 23% indicated it as a choice in cases without the possibility of an alternative (second/third choice), in particular if health, especially the cognitive aspect, worsens. Some respondents (10%) also underlined the option of cohabitation with children. At the territorial level, the nursing home option was more widely expressed in the Marche region (45%), whereas the “home” option emerged for more than half of the respondents in both the Calabria and Lombardy regions (“at home with a PCA” prevails however in the Marche, 28%). Cohabitation with children was indicated almost exclusively in Calabria and Marche, and housing proximity to children was considered a possibility only by four older people living in the Marche region. It should be noted that in some cases, despite a hypothesis being expressed, a certain overall insecurity in terms of future housing solutions was also expressed, with the need to make such a decision with one’s family.

#### 3.2.2. Home, ‘Sweet Home’ and PCA

The qualitative analysis of the answers confirmed the generalised desire to stay at home for as long as possible. Ageing in place, with one’s own memories and social network, is therefore considered desirable and certainly the best solution for most of the interviewees.

*It is better to stay at home, where I have a memory of everything. (MAR_15)*

*I am too attached to this house (...), I hope my family will leave me here. This is my wish, what I told them. (MAR_10)*

*For me, staying in this house is the best solution. Where should I go? I do not want to go anywhere, this is my home, my city! (CAL_31)*

*I want to stay here [at home]. (...) If I go out for a walk, I can talk with persons I know, I can greet them, I can have company. (MAR_8)*

Some of those who already have a PCA underlined the desire to stay at home with this personal assistant (cohabiting or not), and to be able to interact with her/him.

*For me, to age in this home is the best solution (…). Here I have a PCA, I have all what I want, if I feel bad, I call her. (CAL_39)*

In particular, some respondents with a daily or nightly PCA, expressed hope for a future cohabitant solution with the financial help of their children.

*Right now, I only need help [of PCA] at night. If I should need more support even during the day, my children will provide it (…). They will help me for having a living in PCA. I want to stay in my home, I do not want to go anywhere else. (CAL_38)*

Even in cases where a PCA was not yet hired, some respondents considered constant help from an assistant as a concrete possibility to ensure ageing at home, so as not to place undue stress on their children for caregiving.

*Yes, I would prefer to stay in my home, and to find a solution, a support to continue to stay here. PCA is a solution, also to relief children. (MAR_18)*

For example, an older woman without a PCA, expressed that a support could be a good solution providing she ages without cognitive problems, and that family members supervise the care work of such assistant.

*[To hire] a PCA? (…). If my head fits as I do now, it would be fine too! If I freak out, a relative should always monitor her work. (LOM_32)*

Among those who do not have a PCA, some do not consider this assistance a realistic solution since they believe it to be an expensive option.

*I cannot afford a PCA, because he/she wants at least 600–700 EUR a month. I take only 1000 EUR. What could I do with 300 EUR left? (CAL_16)*

#### 3.2.3. Nursing Home: Absolutely Not!

The nursing home was, by 70% of respondents, perceived as oppositional to ageing in one’s own home; therefore, it was rejected, due to being considered as a place where older persons are not listened to if they need help, but also for economic reasons.

*Who takes me in nursing home? Economically I cannot afford it (…). Moreover, I know that in such a place, when one complains, no help is available. (MAR_14)*

The option of a nursing home is also rejected and is perceived as leading to a loss of freedom and control over one’s own existence, as a place where strict rules must be followed and where older people run the risk of living a flat life and not doing what they would like.

*I will never go to a facility! Oh no, because at home I feel free with everything. There you are forced to eat at a scheduled time. (…). You cannot go out when you want, you cannot smoke a cigarette. (LOM_26)*

Some interviewees also fear becoming victim to mistreatment. In fact, newspapers and TV often provide a very negative picture of the assistance provided in this type of facility.

*I do not think I will go to the nursing home (...). In TV I see how older people is badly trated there. Eh, no, no, no, I really do not want go there. (MAR_22).*

For some, the nursing home even represents an ineluctable path towards death, both physical and emotional, with the risk of losing the will to live.

*The nursing home is the death of older people. If one wants to die first, he/she goes there. It is terrible, terrible. There you must leave with strangers you do not know (…). You are immersed in your memories (...), and you turn off. Gradually you turn off. (CAL_7)*

#### 3.2.4. Nursing Home: Maybe

As for the nursing home option, the attitude of some older people is less clear-cut. It remains a solution to be generally avoided, and eventually accepted only as a last chance (second/third choice) in the absence of better alternatives and help, when older people are no longer able to carry out any daily activity and take care of themselves. However, this choice is prefigured more as an action suffered than adopted autonomously.

*As long as I can, I will stay here at home (…). Then, if I become dependent, I will go to a nursing home (…). I think it is the only solution. If I cannot do anything anymore, I cannot remain at home. (MAR_13)*

The nursing home is considered as a last resort even when cognitive impairments occur.

*When I no longer understand anything, I think they [children] will put me in a nursing home, but as long as I stay with my head, I remain here in my home. (MAR_5)*

#### 3.2.5. Nursing Home: Accepted

In the very few cases in which the nursing home is accepted, and a more positive perception (first choice) of it is reported, this can be linked to particular factors, such as the following: realities of high quality and cost, even operating abroad; the constant presence of medical personnel; and the possibility of relieving family members, especially children, from the burden of care.

*I do not know what the future holds for me. Maybe I will go to a facility, but not in Italy. Maybe I should go to Switzerland, where there are many nursing homes of high quality. (CAL_12)*

*Children have their own interests and commitments (...). I do not want to disturb them, absolutely! (…). I prefer the nursing home. There is always the medical doctor! Then, when it is time, they bring you your medicine (…). (LOM_36)*

#### 3.2.6. Cohabitation and Proximity with Children

There are very few cases in which the eventuality of going to live with children was actually contemplated, at least as an alternative to a nursing home, and with the need to sell one’s own home.

*If my health deteriorates, of course I should sell my house and I could even retire with someone (…). I would go to live with my daughter. (MAR_5)*

Older people expressed a certain reluctance towards cohabitation with children also because it would imply moving elsewhere, where they do not know anyone, and having to give up their freedom, even if they are living with family members.

*My son wants keep me with him out of my city [another town in the province]. Here, if I open the door, someone always passes by and greets me. In other places this cannot happen because there nobody knows me. (CAL_35)*

In one case, future cohabitation with one of the participant’s daughters was refused, since it was already experienced in the past (due to the need for assistance after a fall and related fracture), and perceived as a negative period.

*I had to stay in my daughter’s house for five months [due to a fracture], I could not take it anymore! (MAR_30)*

Only four older people (in Marche region) hope for a future geographical proximity with children, who could take care of them.

*I cannot live alone. I cannot remain in this home (…). A small apartment, close to my daughters, would be enough for me. (MAR_9)*

#### 3.2.7. Further Housing Solutions

There were also two proposals similar to ‘co-housing’ in the Calabria region, which are indicated as further alternatives to the impractical expense of nursing homes. For instance, an old woman would prefer to age with some friends.

*The best thing would be to live with some friends in the same house, all together. Eh, but it is not easy to be realised. (CAL_7)*

Three older people even contemplated the possibility of transferring directly to the hospital, as this could be both an emergency and definitive housing solution (for health reasons).

*Depending on how [health] things are, if I have to go to the hospital or clinic, I go. (CAL_10)*

#### 3.2.8. “I Am Unsure”

In some situations, although some hypotheses were put forward, respondents were undecided about their future housing solution, since they were aware that they could not decide/choose independently on this, but conversely it would be necessary also to consult their family, especially in cases where they become seriously ill and require assistance.

*If a debilitating disease takes over, we logically cannot decide by ourselves. Then the others [family members] will decide, unfortunately. (MAR_4)*

*If my health were to deteriorate, I would discuss with my children and make the appropriate decision [for an adeguate housing solution]. When there are decisions of a certain importance, of a certain consistency, I always consult my children. (CAL_9)*

### 3.3. Family or Public Responsibility in Caring for Older People

#### 3.3.1. Different Regulatory Orientations

Besides the opinions on future housing solutions, the interviewees were also asked if caregiving for an older person should be a priority task for families/children, or if greater support should be provided by public services (e.g., home care) (Table 4).

The majority (42%) believed that, firstly, family members/children should take care of their older loved ones, if of course they can and are available. This is followed by 32% of those who considered collaboration between/co-responsibility of family and public services as better, with public services supporting both older people and family caregivers. There were fewer respondents (22%) who considered assistance to be a priority/exclusive role of the public sector, especially for older people without a family. The respondents in the North, more so than those in the Centre and South, expressed their beliefs that assistance is a family duty (58%). In the Calabria and Marche regions, 60% of older people desired greater support from public services, alone or with the family, compared to 40% in Lombardy.

#### 3.3.2. The Family, If Any, as First Care Provider

The narratives/quotations confirm and complement the quantitative picture. Among those who argue that assistance for older people is primarily a responsibility of the family, some think in particular that this is an old “general axiom”, and it must be followed as such.

*When I was young, grandparents were not abandoned and they were assisted by the whole family. This is the rule. (LOM_17)*

The family, especially children, if there are any, are therefore the first choice, for emotional/affective reasons, because the family is always considered as the best solution.

*The family is always in the frontline, because the family is in the heart. (MAR_19)*

*Help comes from the family, from children (...). Children should not abandon their parents! (CAL_38)*

In particular, some respondents who reported previous personal experience of family caregiving for their parents, considered this the only feasible option, believing it to be a family role by default.

*I think that children are the first caregivers, because it is right so. A mother and a father take care of their children. If there is the possibility, it is right that children help [the parents], as I did it towards my parents. It is a duty of children. (CAL_6)*

However, when one does not have children, the assistance of kinship in a broad/extended sense seems possible, even though care by children would be better.

*As a general rule, it would be nice to be assisted by the family, but in my case, I do not have a family and children of my own (…). I could have support from grandchildren [daughters of sisters/brothers], but I would have preferred more to be assisted by a child. (LOM_29)*

The family also helps older people to feel more calm and secure, whereas when care is provided by other persons, by “strangers”, one has to adapt.

*I am convinced that older people must be cared for by relatives, because I think it is much better having support from loved ones than from strangers. (MAR_14)*

However, some respondents highlight that, in this respect, there are pre-conditions to be considered. The family caregiving can be accepted if there are several available relatives, but without obligations, because most of them work and in turn have their own families. In some cases, it is also a gender issue, e.g., women are considered as better than men as caregivers.

*Well, if there is an extended and available family, it is clearly nice that relatives take care of their older loved ones! (CAL_7)*

*Children are better, but it is clear that they have their jobs, their families and own babies. They help, but I cannot force them. (MAR_10).*

*I would prefer a niece, a cousin, I do not know exactly. However, a woman in any case (...). I went to the hospital and they made fun of me because I did not want male nurses to clean me. (LOM_1)*

In addition, the family must be united/cohesive and capable of caring, otherwise a “stranger” is preferred.

*If a family is unable [to assist], is not smart enough to understand which are the needs of the older parents, then it is better to be assisted by other persons out of the family. (LOM_13)*

There are those who attributed to the family also a controlling role for PCAs. Conversely, other more intransigent interviewees believed that it is almost a shame to resort to a PCA if there is a family.

*[When a PCA is hired] Family members must always be present, because there are good PCAs who assist well and others who do it exclusively for money (…). Family members must be present, and be vigilant. (CAL_18)*

*I think that older people must be cared for by their families, but unfortunately currently there are many PCAs in this respect. This is not good. Everyone should take care of their own older family members. (CAL_30*)

#### 3.3.3. Family First, but with Public Support

Attitudes have changed, and families in Italy are no longer those (extended) of past years, when all members lived together and helped each other.

*Once, when families were extended, one could exchange/alternate in supporting older relatives. Now the families are small and it is no longer possible. Treating a sick person alone, without help, is a heavy thing. (MAR_39)*

In light of this, a family–public support network could work better. Family would be first, but with the assistance of public support.

*The family is primary, but public service is indispensable. (MAR_4)*

*The care from the family by civil law must exist, but it is also a task of social structures, of public services. (CAL_12)*

Furthermore, children often need to help themselves, because they have their own family (spouse, children) and work commitments. Public services must therefore also support the caring family.

*Families today are not always available, they have jobs. They need someone from public services who can support/relief them. (MAR_36)*

In particular, it would ideally be desirable to have public services for physical/personal support (“hard” care), and family and children for company and affective support (“soft” care). Family should therefore be raised and integrated by public services in the former tasks.

*The presence of children is good for what concerns the affective side, the emotional closeness. It is acceptable that children visit parents or hear them on the phone frequently, but it is heavy to support them for the activities of daily living. (MAR_18)*

However, for one respondent, the public integration of family assistance should only concern health-related aspects. In another case, it is defined as allowance, e.g., as a provision of IA, even though it does not seem to be enough.

*The first support is the family, when needed (…). The public service should intervene for health needs and medical care. (CAL_20)*

*The family and IA (...). This association could work, even though the latter sometimes is not sufficient for medicines and treatments that are needed. (CAL_27)*

This underlines how, at times, previous personal experience of (heavy) caregiving makes one feel the need for public support for their own family members when being cared for, as they cannot manage such a difficult task without assistance.

*My children cannot help me (…). I do not want they live the [heavy] caregiving experience I had when supported my aunt, my mother-in-law, my father. (CAL_35)*

With public help for the family, perhaps older people themselves would feel less loneliness at home.

*Family and public service. The one does not exclude the other. Children work and cannot be always available. When sometimes I stay with my daughter in Rome, she goes out to work at 9.00 am hour in the morning, and comes back at 8.00 pm in the evening. All day I am alone and dumb like a mummy! (CAL_1)*

However, not all seniors accept help by someone other than their family.

*There are also some seniors who do not want anyone in their home for assistance, other than the family. (MAR_1)*

#### 3.3.4. Public Service as Priority or Exclusively

Some respondents think that public services should intervene firstly as a “general rule”, as a priority or with exclusive help, for older people who have no family/children, or for those who have children who cannot assist them.

*There must be greater public attention for older people without children, or other relatives, who cannot assist them. (CAL_7)*

*Public services must help older people, since family members have their own families. (MAR_38)*

The role of public services was considered as central even when seniors do not have good relations with their families. In fact, a couple of responses from older people with little contact with their family indirectly suggest the influence of negative or “loose” family relationships on the choice of the public service as a priority care solution, and indicate dissatisfaction in the stereotyped icon of the loving family, as usually constructed by the collective imagination.

*Children cannot be relied upon because they abandon their parents in nursing homes. It does not mean anything to have children! So do not tell me the story of the lovely family. The family does not exist! If you have a lot of money, then you may be treated well. This is the law of life. (CAL_15)*

#### 3.3.5. Does the Public Service Work?

Regardless of the caring option indicated above, the idea that public service provision is lacking seems to be a common denominator in some situations (data not in the table).

*Family members need to assist their relatives because public services do nothing. (CAL_24)*

*The Italian State just abandoned seniors and does not help them. This is not the case abroad, where the State intervenes. (MAR_31)*

### 3.4. Future Housing Solutions and Other Dimensions

From the examination of the relationship between future housing solutions and other dimensions, some links emerged (Table 5).

Men, slightly more so than women, want to stay at home, even with a PCA. This combined housing solution was also preferred by those who are currently supported by the family (23%), and those who already have a PCA (26%), especially if they reported a positive experience in this respect.

*My children help me, but I have a PCA every day. She’s very good, it’s a positive experience, that I would also recommend. (MAR_24)*

The nursing home is preferred above all by those who live alone (33%), especially if they are tired of this situation of loneliness.

*At least there [nursing home] there is someone who says goodnight to you. Do you have an idea of what is it like to eat alone every day for years and years and years? Going to bed alone for years and years and years? (LOM_14)*

Mobility seems to have little or no influence on future housing choices. However, the level of functional limitations, although it does not greatly affect the choice of nursing home, seems to be more related to the preference for the home when moderate/high, and for a PCA in the home when mild/moderate, but also very high in some cases.

### 3.5. Family or Public Care Responsibility and Other Dimensions

From the analysis of the relationship between family or public responsibility in the caring for older people, and other dimensions, other links emerged (Table 6).

Those who are already supported by their relatives, in contrast to those supported by public services, strongly believe that caring is a family responsibility (40%).

*I have my sister, if I need something, she is available. If there is a need, it is better to have help from the family. (MAR_13)*

Moreover, the family represents the ideal assistance network slightly more for respondents who can move also outside the home, albeit with help, and for those who have less difficulty in carrying out daily activities, as a result of mild and moderate physical limitations. The family–public service dyad prevails instead among seniors with higher limitations (moderate/high), and among those with even higher limitations (high/very high) it emerged that public intervention should be the main actor in caring for older people, so as not to disturb the family.

*More public services [should assist seniors]. I do not want to disturb my children, who work a lot, and have their own families too! Why should they take care of my several health problems? This does not suit me on principle. (MAR_22)*

The family–public service binomial also prevailed among women (36%) and those with a PCA (37%), whereas the priority role of public services was indicated above all by men (33%), and by those who live alone (23%), especially those without family/children.

*In my opinion, the family should help those who have it. I do not have a family, thus the Municipality, the Region, should help me with public services. (MAR_34)*

Even those who already received help from public services seemed to indicate the need for priority/greater responsibility in formal support for caring for older people (23%). Additionally, they require more intensive public assistance.

*Public services must intervene firstly, and much more. I need so much and what SAD is delivering is too scarce. I would like more help. Four hours a week is not enough. (CAL_8)*

### 3.6. Future Housing Solutions and Family/Public Responsibility of Caring for Older People

Finally, from the examination of the (only quantitative) relationship between preferred future housing solutions and opinions on family/public responsibility of caring for older people, further links emerged (Table 7).

Respondents who preferred ageing at home, in great part consider taking care of older people a responsibility of the family (52%). Those who preferred ageing at home with a PCA largely attribute this task to public services (42%). Seniors who proposed also ageing in a nursing home, think above all that caregiving is the responsibility of the family, or of the family with the support of public services (33% for both).

## 4. Discussion

The aim of this study was to explore the opinions of frail older people with physical limitations living alone in Italy, with regard to both preferred future housing solutions and assistance, and regulatory orientation on care responsibilities of family and/or of public services. Our results showed overall that the majority of seniors preferred ageing at home, and consider that firstly family members/children should take care of them. However, preferences and opinions are often mediated by the possible/realistic care solutions, and various positions emerged within this general picture, in addition to some regional differences.

### 4.1. Future Housing Solutions

Our results are on the whole in line with those in the previous literature, especially regarding Southern European countries such as Italy [43], thus indicating that the majority of older people prefer to age in place/in their homes, at least even with a PCA, with little intention to consider moving elsewhere, e.g., to a nursing home or going to live with family/children [70,71,72]. Other authors [73] more specifically found that in later life, 37% of individuals would prefer to live independently at home. If this option is not possible, 17% would like to be supported in their own home, and only 9% could accept moving to a nursing home.

#### 4.1.1. Ageing at Home, at Least with a PCA

For frail older people, their own home is indeed a place that signifies memories and freedom, comfort and safety, where they can continue (in some way) to carry out the activities of daily living and maintain their identity [36], with ageing at home thus implying both functional and emotional dimensions [74,75]. According to Martinelli and colleagues [44], Italian seniors show a strong attachment to their current home, habits, social network of contacts and friendships, which also emerged in the projections regarding their future housing solutions/arrangements. The possibility of ageing in one’s own home and neighbourhood can also improve quality of life [76,77]. The option of ageing at home with a PCA was moreover expressed by some respondents who considered such a support as the concrete opportunity to age in place. Several authors point out that the availability of receiving support at home, at least through help for domestic work, allows older people to age in place [35,78]. In particular, a PCA is usually hired when seniors have health problems and require continuative help and nightly monitoring, but also in cases of widowhood [21].

However, some interviewees considered hiring a PCA to be possible if the related costs are low, and if there are relatives who can monitor/control the work of this kind of support. Studies in the literature also report that the recourse to a PCA is greater when the family is present often enough. Probably, in these cases, the family is considered to be “complementary” to such care and generates the so-called “crowding-in effect” rather than “crowding out”, and a PCA does not substitute for family members, who also retain an important management role and provide oversight for the work of this figure [79,80]. This also highlights that, for some children, a caring responsibility means mainly providing more adequate care for loved ones, and managing/supervising such care. No respondent expressed/specified the preference to stay at home with help from public care services, e.g., SAD, probably indicating that in this circumstance the control of a family would not be available anyway, since the SAD is often granted (moreover with few/insufficient weekly hours) to those without economic and family resources. As a result of the eligibility rules for accessing this service, its main users are indeed older people with overall frailty (functional/physical and relational/social/familiar) [22].

Further respondents specified that PCA is useful for ageing in place and to reduce the stress placed on children associated with caregiving, and if older persons have no cognitive problems, even though the high cost of this support represents a problem. Costa [81] indicated in particular how this personal help solved the discrepancy among the increasing care needs of seniors, scarce provision of public services, and low and decreasing availability of family caregiving. Moreover, the presence of cognitive impairment, hampering the acceptance of help from a PCA, could be related to the fear of being a victim of possible abuse/mistreatment by this figure. Regarding the fear of abuse, previous literature provides evidence of older people sometimes being mistreated by privately hired PCAs (both physical and psychological abuse) [82]. The main risk factors for elder abuse are indeed related to the specific characteristics of the victims, and include, among others, cognitive impairment, dementia, poor mental health, depressive symptoms, and physical disabilities [83]. Previous studies in the literature also stressed the issue of the high cost of PCAs, impeding their involvement in caregiving for older frail adults with poor financial conditions [21].

#### 4.1.2. Ageing in a Nursing Home

In our study, the nursing home was above all rejected as an alternative housing solution vs. staying at home, being perceived as a place without freedom and social relations, with strict rules to be followed. For some respondents, the nursing home even represents an ineluctable path towards death. Additionally, studies in the literature report that moving to the nursing home strongly impacts the everyday life of seniors and their relationships with family and friends, making them socially isolated, alone, and leading to depressive symptoms [84]. A study explored in particular the role of loneliness and depression as risk factors for suicidal ideation among residents in nursing homes, and found that about 20% of them experienced such a thought [85]. Some interviewees also reported that, as was the case for PCAs, the nursing home is rejected for economic reasons, as highlighted by Chimento-Díaz and colleagues [70], who associated the degree of institutionalization with economic issues, in addition to the level of dependence of seniors.

Furthermore, interviewees reported fears of being victims of mistreatment in nursing homes, given the negative information reported in newspapers and TV in this respect. Literature and media indeed often reported abuse within facilities for older people, including lack of hygienic conditions, insufficient care personnel, or expired medications and malnutrition [82]. Nursing homes were reported as a possible future housing solution, even though as a last option, once the respondents in our study develop functional limitations and have no other recourse for help, e.g., by family. Filipovič Hrast and colleagues [35] underlined how childless and single people are more likely to move and more commonly live in institutionalised settings. Relocation decisions are in particular linked to the location/proximity of the closest relatives, e.g., children. The absence of family members living close could thus lead seniors to move from their home [86]. Moreover, differently from PCAs, the nursing home is considered as a last resort when cognitive impairments occur, as certain mental conditions could diminish the feelings of sadness associated with being in such a place and make this housing less painful.

Additionally, other authors [45] found that institutionalization could be an alternative housing solution when older people become increasing frail, with decreasing cognitive ability, and thus requiring specialized care. Older adults with neurocognitive disorder/dementia indeed have higher needs and risk for institutionalization, and architecture experts often focus on residential facilities when designing housing solutions for them [87]. In a few cases and in particular circumstances, the nursing home was accepted, even as a first/voluntary choice, for instance when high quality settings are available, even abroad. Some authors [88] found that older residents in care facilities, especially when free from cognitive impairment, perceived a good life in such settings, due to the high quality of both staff and life conditions experienced there, in addition to decreasing the sense of loneliness and providing interaction with other residents. Rijnaard and colleagues [89] specified, however, that in this respect, factors such as the maintenance of one’s own habits and available private space are fundamental. Other authors reported a more positive attitude of seniors towards the nursing home as an option providing a simplification of daily life, even this depends on their financial resources [44].

Another reason for accepting this housing solution is the constant presence of medical personnel, thus increasing the sense of safety and security of the seniors interviewed. This is also reported in the previous literature, with the specification that seniors living at home are more likely to have hospital admissions than those in institutions [90], and that a quarter of admissions of older people in residential facilities seems associated with their inability to self-administer drugs [91]. Moreover, in another study [92], senior residents showed a decreasing prevalence in both polypharmacy and complex therapies, with regard to those ageing at home. Our respondents also referred to the acceptance of moving to a nursing home to relieve family members of the burden of caring. Previous authors associated home care with a greater burden on family carers [90], and stressed how support from children is beneficial to older people when it is moderate, since if it is excessive it could make them feel guilty of being cared for [93].

#### 4.1.3. Ageing in Cohabitation or Proximity with Children

Regarding a possible future cohabitation with children, older people expressed a certain reluctance since this option would imply both moving to another city/neighbourhood where inhabitants are unknown, and losing their freedom/autonomy. The literature highlights how the characteristics of neighbourhood impact daily activities such as walking, shopping, and social relations, including the feeling of security and social inclusion. Conversely, when their habitual neighbourhood changes, the well-being of seniors seems affected [94]. The literature also supports the finding that co-residence in a relative’s home is not desired when older people perceive this care arrangement as decreasing their independence and autonomy [95], and moreover when they do not want to become a burden for the family [96]. Other authors [36] provide evidence that older people are not always convinced of the benefits of living with relatives, since living alone positively allows autonomy in making one’s own decisions, and living with working children does not necessarily help to avoid feelings of loneliness or needing external help.

Additionally, Meggiolaro and Ongaro [97] found that close contact between older parents and their children, as occurs when co-residence is a forced mode of cohabitation, does not improve the quality of life of seniors to a great extent. Only four older respondents in our study consider a future/possible geographical proximity with children, in cases of need. Some authors [98,99,100] indicated that this circumstance allows for family caring, and nursing homes or formal care are less involved when at least one child lives nearby, especially for parents with functional limitations and living alone. Choi and colleagues [99] also found that prediction for seniors needing help to remain in a community is 32% when there is a co-resident child, 26% when a child lives nearby, and 21% if there is no close child. Previous studies stressed the influence of the proximity of parents and children on both the nature and frequency of contacts [101]. Overall, it is to be considered that a decline in both co-residence and the geographical proximity of older people with their children, in addition to increased participation of women in the labour market, have reduced the availability of potential informal carers [102].

#### 4.1.4. Further Consideration on Future Housing

Finally, only two respondents cited a possible form of co-housing, that some authors highlight as a good option for seniors without severe care needs, and who would age with the company of age peers, but do not want to move to a nursing home, thus potentially reducing loneliness [103]. In Italy, few examples of co-housing are currently available and known of, e.g., The Solidarity Cohabitation Program of Auser (volunteering association) [104], which represents the first experience in this country (Tuscany region, 32 Municipalities in the province of Florence) of the inclusion of senior cohabitation as a new way of responding to multiple needs in the local welfare system, with a reduction in inappropriate services use and related expenditure, through personalization of the intervention. It is also worth noting that, in some cases, respondents are insecure about their possible future housing solutions, thus needing to fix the issue with the family, and depending on the related concrete caring possibilities. Previous literature [105] found that family composition, and current relations with both children and grandchildren, affect preferences and perceptions of future care arrangements.

### 4.2. Family or Public Care Responsibility

#### 4.2.1. Family as First Care Provider

A large percentage of the respondents believe that the family, especially children, if there are any, should have the first responsibility for caring for their older relatives needing help, for emotional/affective/security reasons, and it is considered as the best solution when compared to assistance provided by “others”. This is in line with the Italian family culture/model and with the family-care regime, that elects relatives as best providers of LTC, especially for frail older people, when compared with public services [106]. Family carers can in fact be an effective alternative to public support [107], although there are few current public policies available for caring families [108]. In particular, in Southern European countries, such as Italy, there is a legal recognition of the care responsibilities of families, especially as a duty/obligation of children, but there is not adequate public support, whereas in Northern European countries this attribution is limited, with a greater role of public services [45].

However, respondents in our study specified that, in any case, a female caregiver is better than a male one. This mirrors the traditional care context, especially in Southern Europe [109], where usually daughters, more than sons, take care of their older parents, also because they are considered more naturally inclined in this regard [110]. The family responsibility for caring also involves control of/supervising the work provided by a PCA, as already discussed above, with regard to a possible ageing in place with this caring figure. It is moreover interesting to highlight that some respondents considered caregiving a family role by default when having previously experienced family caregiving for their parents. This finding once again highlights the Italian tradition of family caregiving, and the overall intergenerational solidarity/help exchange pattern in ageing societies [111].

In other situations, however, some interviewees conversely expressed the need for public services to support their family due to the personal burden experienced when caring for their family. The literature addresses this issue, confirming that caregivers have different views on caring responsibilities depending on their past experience, and specifying that, especially when they are burdened, a greater responsibility for the personal and nursing care of older people has to be placed on the government/public sector. Thus, a less heavy/hard personal experience of assistance is associated with perceiving a minor involvement of public services in terms of caring assistance [112].

#### 4.2.2. Family and Public Services

When older people suggest that care responsibilities should be shared by both their family and public services, they argue that current families are small with few potential carers compared to the past, and moreover, caring children themselves need help, in particular due to work reasons. Thus, public services should support both carers and cared-for people. In the current context of Italian society, women, once seen as traditional caregivers, are now more involved in the labour market [23], and the size of families has decreased, with the average number of components decreasing from 3.35 in 1971 to 2.29 in 2019, due to economic and social transformations such as the decline in births, and the progressive ageing of the population itself [113]. Additionally, Lüdecke and colleagues [114] stressed the necessity for appropriate public health and social care support for family carers when extended family networks cannot provide the required assistance. In particular, respondents in our study indicated public services as providers of personal/health support, and family and children as the main providers of emotional support. These results are almost in line with those from other authors, who highlighted how older people consider the government as responsible for personal/nursing care, and relatives for social support and household activities [112]. Some European approaches (e.g., in England, France, Germany, and the Netherlands) recognize family caregivers as formal “co-workers” or “care providers”, with formal care provision cooperating with informal care, and ensure dedicated payments, contracting, and training opportunities [115]. In Italy, there is not yet a national law (apart from some regional initiatives, e.g., Emilia-Romagna and Abruzzo) that establishes the rights and duties of caregivers as official care providers within the LTC system. However, it is necessary to consider Stability Law n. 205, of 27 December 2017, to find an initial national definition and relative recognition of the role of family members who assist, with the provision of an economic support fund [116].

#### 4.2.3. Public Services as First Care Provider

Some respondents think that public services should firstly take care of frail seniors, especially when no relatives/children are available. Moreover, children should not be disturbed at all since they have work commitments. These findings can be found also in the previous literature, pointing out how both older care recipients and their family carers, consider the government as the main agent responsible for care provision [112,117], since employment negatively impacts the actual possibility and willingness of people to act as caregivers of their loved ones [118], especially when initiatives for supporting working carers are not well developed [119]. Our results further highlighted that relations between older people and their families affect the perceived caring role of public services of the former. Thus, care expectations of seniors can be influenced by good or bad relationships with family members [38], habitual intergenerational contacts [120], and the family’s overall history [80].

When contact with family is loose/deteriorated, older people indicate public services as a priority care solution, since they cannot place trust in the care of a loving family. Wise [121] characterised underlying concepts such as filial responsibility, gratitude, and reciprocity as ‘romanticized’ theories for attributing care of older parents to adult children, with the family acting as a “haven in a heartless world” [122], whereas in reality, at least for some individuals, families are not a source of love, comfort, and support. Finally, regardless of the different caring responsibilities indicated above, some seniors expressed the opinion that public services are lacking/not efficient, and this reflects the awareness that in Italy the formal care sector is inadequate, especially for older people, as indicated in the introduction of the paper. Existing social-health policies in a country may thus guide preferences, with trust placed in public care responsibility when LTC systems are robust, and vice versa [117].

### 4.3. Future Housing Solutions, Family/Public Care Responsibility and Other Dimensions

Regarding future housing solutions, men prefer to age at home with a PCA, thus indicating how they usually, more than women, need help for managing daily housekeeping [123]. Additionally, seniors who are currently supported by their family, and those who already have a PCA, prefer ageing at home, indicating the presence of family and private help as positive assets that may permit ageing in place rather than moving to a nursing home [27]. The latter housing solution is conversely preferred when seniors live alone without the support of a PCA, and this presents evidence of how ageing in place can lead to isolation and loneliness [124], whereas in a nursing home seniors may at least interact with other older people and feel less alone [125]. Moreover, the functional limitations of respondents in our study do not greatly affect the choice of moving to a nursing home, whereas previous findings support a positive link between low ability in providing activities of daily living and access to residential care [53], probably indicating the gap between the preference of frail seniors and actual housing solutions, often decided by the family. The *Behavioural Model of Elderly Migration* by Wieseman [126], specifies that the overall process of relocating seniors elsewhere from their home, is influenced not only by triggering events, such as functional decline and related needs, but also by personal resources, such as available income, not included in the analysis, due to the wide range of income classes included in our study, since interviewees often did not refer their required monthly punctual income.

Regarding family or public care responsibility, seniors who are supported by relatives, more than those supported by public services, consider caregiving first of all a “family matter”, probably as they hope to continue receiving this help in the future, in the light of cohesion, solidarity, and reciprocity among family members, as intergenerational transmission of moral values [127]. This is also the opinion of older people with low functional limitations, whereas those with greater difficulty in carrying out daily activities are more oriented towards family–public service cooperation, or even indicate receiving support only from the latter. These results suggest that even though a preference for moving to a nursing home is not greatly linked to the level of autonomy (as stated above), when reaching an ideal care responsibility, seniors do not want to burden their family, especially children, with caregiving when severe health/physical problems exacerbate. Pinquart and Sörensen [128] relatedly highlight how older people indicate the option of the exclusive use of informal/family support, in case they have short-term care needs and, conversely, the option of the exclusive use of formal support in cases of LTC needs.

The gender issue emerges in the prevalence of family–public service care responsibility among women, and mainly public/formal care among men. Previous literature also points out that female caregivers have larger social networks and more available informal support, whereas male caregivers are more inclined to seek external help [129], and thus they place a greater responsibility on the government for caring for seniors [112]. The priority role of public services is also determined by those who live alone without help from a PCA, and those who already have this help. Probably, in both cases, there are no existing/available family caregivers. Some authors indeed underline that, with regard to older people living alone, household composition can predict the use of public home help services [130]. Finally, when reading opinions on future housing solutions and family/public responsibility when caring for older people, three main points emerged. Seniors desired ageing in place, largely believed that caring is a responsibility of the family, and supported the combination of home–family care.

Those who age at home with a PCA, assign caring as a task mainly of public services, thus indicating a general preference for the home–external help combination, the latter coming from a personal paid assistant, but ideally to be provided for free or partly free by the formal sector, probably also due to affordability. Seniors who proposed (also) ageing in a nursing home, place the caring task on the family, at least with some form of public integration. These findings seem to support a possible overall trade-off from ideal home care vs. a necessary care home, in order to relieve the family and in particular not to disturb the children, as reported by some interviewees. In summary, as stressed in the literature, the opportunity to age in place, at least with support, seems a theme that runs (more or less evidently) across the opinions of all the participants, and the family is always (more or less explicitly) called into question [1,131].

### 4.4. Regional/Territorial Differences

At a territorial level, in our study, the nursing home option emerged as more preferred in the Marche region, and home care was preferred in the Calabria and Lombardy regions. Moreover, the respondents in the Northern region, more than those in the Central Southern regions, think that assistance is a family task. In the Calabria and Marche regions, the majority of older people in each, compared to Lombardy, think that greater support should come from public services, alone or with the family. Currently, however, older Italians are assisted by the family mainly in the South-Centre, whereas SAD is mostly available in the North-Centre [22]. Overall, these findings seem to suggest that seniors in the North desire the increased “implementation” of traditional family care in the home, which is currently low, whereas seniors in the South seem to prefer “revisited” care in the home, with the support of public services, which is currently scarce.

However, the lower expectation of seniors with respect to the caring role of the family in the South could be linked to the realistic awareness that this support is not sustainable in the future, also as a result of the need, especially of children, to emigrate towards other contexts to work and survive, hence reducing their care function [132]. In this respect, some authors, e.g., [133], highlight that the alternative is sometimes the transfer from South to the Centre-North of parents who are older and no longer able to live alone, and are therefore forced to reach out to their migrant children. In the Centre, the opinions of seniors interviewed revealed the aim to benefit from public services, e.g., SAD, currently provided but deemed as not sufficient, thus presenting the possibility to move to a nursing home if needed. This could represent a sort of shift towards a progressive “reinforcement” and “substitution” of family care in the home, in a “middle” part of Italy where public services are provided but not as in the North, and family care is present but not as pronounced as in the South.

### 4.5. Limitations and Trustworthiness of the Study

The study has some limitations. The survey was carried out only in three Italian regions, and moreover without including respondents from metropolitan areas. The definition of frailty is limited to age (65 years and over), living alone without cohabiting relatives, and the presence of functional limitations, thus needing support. The cognitive assessment of interviewees was provided with the help of recruitment channels, and information from respective families. The low number of PCAs included in the study may result from the exclusion of older people with high physical and cognitive limitations (in order to be able to answer questions independently), who represent individuals often supported by these private assistants. Finally, the percentage values in tables should be interpreted with caution, since related absolute values are sometimes very low. Despite these limitations, the trustworthiness of our study, in particular of the qualitative analysis, is based on the criteria of credibility, transferability, dependability and confirmability, as indicated by Lincoln and Guba [134].

The credibility is based on the use of a topic guide containing inputs from questionnaires applied in previous studies on frail older people [55], and on peer de-briefing sessions among multidisciplinary researchers, in order to define protocol, data collection-analysis, and discuss findings. The transferability of the qualitative analysis [135] was satisfied by a deep literature review [136] and the analysis of several studies on the topic [27], which were crucial for building the initial framework [23]. The dependability and confirmability of results, using replicable methods, were supported by a detailed study protocol (approved by a Bioethics Committee), with several specifications regarding data collection and analysis. For more details on the limitations and trustworthiness of the “IN-AGE” study, further information can be found in a previous publication [22], from which these aspects are partially adapted.

## 5. Conclusions

The exploration of the opinions of frail older people on possible future housing solutions and care responsibilities provides an articulated and interesting picture that can be of help for understanding and elaborating the gap among available care, preferred care, and different regulatory orientations, especially in the light of an ageing population with both an increase in life expectancy and need of support, with a greater preference for community care. The majority of frail older people with physical limitations, living alone without cohabiting relatives in Italy, and especially those currently supported by the family, would prefer ageing at home, at least with the support of a PCA, thus maintaining habits and providing continuity in social relationships. They also indicate the family as primarily responsible for caregiving, at least with public support, despite some territorial differences emerging, e.g., more desire of family in the North and of public services in the South, that is an overall context opposite to the current care situation of seniors in these parts of the country. However, overall, there are several, also opposite, nuances. Respondents who had a previous personal experience of caregiving for their parents, in some cases consider this as a social rule; in other situations, they feel the need for public support for their caring family members. Additionally, the gender perspective emerged throughout the findings, since some respondents expressed a clear preference for ageing in place with the support of a female caregiver, as they are considered as naturally and traditionally inclined for this task.

Moreover, some interviewees consider the nursing home as a place of loneliness, whereas others feel more alone in their own home. The possibility of future cognitive problems drives the choice towards a nursing home, because older residents with such problems are less aware of the disadvantages of such housing, whereas when a PCA is hired, it is better to have a “quite good head” in order to be aware of the assistance received. PCA and nursing homes are both considered costly for some seniors, and both solutions present risk for elder abuse/maltreatment. Caring for older people depends on several factors, including the concrete provision of formal care services in a country [137]. Additionally, a gap emerged between where older people would “ideally” age, and where they will “realistically” have to age, and in turn between the desire to satisfy their needs “in the family” and the necessity (but not the preference) to seek help “outside the family”, especially if the level of physical limitations is considerable. Thus, insecurity in this respect is referred to in some cases, indicating that it is extremely complex/difficult, for older people to imagine their future situation. In the end, the effective caring possibilities of the family, if any, in addition to the quality of relationships with relatives/children, and the available public services, play a key role, either providing or not providing the intergenerational solidarity. Sometimes, the interviewees, rather than expressing a preference or a theoretical opinion, responded as influenced by their current care situation, that in turn could impact their possible/preferred future care context. The overall analysis thus focuses on how, in later life, older people would prefer to age in place with family close by, and if this is not possible, especially if children are not available, they resign to opting for different supports. A good solution/compromise could be a complementary model in the LTC provision for older people, where formal/public services and informal/family/private care are integrated.

New housing concepts should thus be developed, bridging the distance between ageing in place and in nursing homes, by focusing on interventions based on a deep evaluation of the needs and desires of both seniors and their respective families [87,138], in particular by supporting family caregivers and redeveloping residential facilities, with higher performance and management standards, and the promotion of alternative housing measures, e.g., co-housing [139]. For this purpose, the preferences and reasonings of seniors should be considered, and investigated in depth with further research, paying careful attention to involuntary movers and involuntary stayers from/in their own home [87], but also to overcome the usual binary position, i.e., ageing at home or in a nursing home, in order to explore possible new directions of moving [35] and providing useful insights for policy makers. More research is also needed in order to better explore and compare how the gender equality issue is going to be addressed in Europe, especially with regard to the care of older people, in the light of the increasing participation of women in the labour market.

Our findings should be interpreted in the light of some limitations of the study, since a more articulated definition of frailty (also social/environmental aspects) and the inclusion of respondents with severe functional limitations (e.g., bedridden older people) and from metropolitan areas (where nursing homes are more widely available and of a higher quality), could reveal different preferences and opinions in older people. Moreover, the inclusion of more Italian regions in the survey could have led to higher absolute values, thus allowing for a more informed analysis in this respect.

## Figures and Tables

**Table 1:** Overview of macro-categories, sub-categories, labels, and quantitative items.

Macro-Categories	Sub-Categories	Labels for the Analysis	Quantitative Items (N = Number) ^1^
**Future housing solutions**	**Preferred future assistance**	Home	N. respondents reporting the main preferred solution(s). In addition, some specifications: - N. respondents with PCA reporting Home with PCA - N. respondents without PCA reporting Home with PCA - N. respondents reporting Nursing home as first choice - N. respondents reporting Nursing home as second/third choice
		Home with Personal Care Assistant (PCA)	
		Nursing Home	
		Cohabitation with children	
		Proximity with children	
		Other	
		Unsure/decision to make with family	
**Regulatory orientation on care**	**Opinion on caring responsibility of family and public services**	Family	N. respondents reporting the opinions
		Family and Public Services	
		Public Services	
		Other	

^1^ The aim of this table is only to provide an overview of main categories and labels which were used in the analysis. Numerical values regarding each label are not reported in this table, but in the following 3 and 4.

**Table 2:** Sample characteristics (absolute values/*n*).

Characteristics	Regions			
	Lombardy	Marche	Calabria	Total
**Age Groups (*****years***)				
67–74	9	4	4	17
75–79	7	6	6	19
80–84	10	11	7	28
85 and over	14	19	23	56
**Gender**				
Male	9	8	13	30
Female	31	32	27	90
**Education**				
No title	1	9	4	14
Primary school (5 years)	16	15	24	55
Middle school (3 years)	8	9	3	20
High school (3–5 years)	15	7	6	28
University/similar (3–5 years)	-	-	3	3
**Marital Status**				
Single	7	5	4	16
Married but not cohabiting	1	1	-	2
Divorced/separated	9	2	3	14
Widowed	23	32	33	88
**Living Situation**				
Alone	36	32	25	93
With personal care assistant (PCA)	4	8	15	27
**Mobility**				
Only in the home	12	17	19	48
Also outside the home with help ^1^	28	23	21	72
**Level of physical limitations ** ^**2**^				
Mild	13	12	5	30
Moderate	8	12	13	33
High	10	8	9	27
Very high	9	8	13	30
**Care arrangements/Supports ** ^**3**^				
Family	27	33	34	94
*Children*	*21*	*23*	*27*	*71*
Public service	14	23	6	43
*Home care (SAD)*	*12*	*11*	*5*	*28*
**Monthly income brackets (EUR)**				
Up to 600	5	3	2	10
601–1500	30	30	29	89
1501–2500	4	6	7	17
2500+	-	-	2	2
Missing	1	1	-	2
**Total Cases/Respondents**	40	40	40	120

^1^ Respondent is able to leave the house at least two times a week, if accompanied or with aids (cane, walker); ^2^ The level of physical/functional limitations is based on 12 ADLs-IADLs, two mobility limitations (going up/down the stairs and bending to pick up an object), plus sensory limitations in hearing and seeing. Mild = no activities “not able”, Moderate = one–two, High = three–four, Very high = five or more. ^3^ Main supports are reported (also friends/neighbours and private services in other cases), and more supports are possible for some respondents (e.g., from family and public service).

**Table 3:** Preferred future housing solutions by region (*n*; %).

Solutions ^1^	Lombardy		Marche		Calabria		Total	
	*n*	%	*n*	%	*n*	%	*n*	%
Home	21	53	17	43	23	58	61	51
Home with PCA	4	10	11	28	9	23	24	20
*PCA already present*	-	-	*4*	10	*3*	8	*7*	6
*PCA absent*	*4*	*10*	*7*	*17*	*6*	*15*	*17*	*14*
Nursing Home	12	30	18	45	6	15	36	30
*As first choice*	*4*	10	*4*	10	*1*	3	*9*	7
*As second/third choice*	*8*	20	*14*	35	*5*	13	*27*	23
Cohabitation with children	1	3	5	13	6	15	12	10
Proximity with children	-	-	4	10	-	-	4	3
Other ^2^	3	8	-	-	4	10	7	6
Unsure/decision to make with family ^3^	5	13	3	8	5	13	13	11
Total respondents	40	100	40	100	40	100	120	100

^1^ More solutions are preferred by respondents; ^2^ 2 cases of co-housing, 3 hospital, 2 “It is better to die”; ^3^ Some respondents refer one/more options, but they also state to be unsure, and that it is necessary to decide with the family.

**Table 4:** Caring for older people: family or public responsibility? By region (*n*; %).

Opinions of Older People	Lombardy		Marche		Calabria		Total	
	*n*	%	*n*	%	*n*	%	*n*	%
Family (e.g., children)	23	58	14	35	13	33	50	42
Family and public services ^1^	12	30	12	30	14	35	38	32
Public services	4	10	12	30	10	25	26	22
Other ^2^	1	3	2	5	3	8	6	5
Total respondents	40	100	40	100	40	100	120	100

^1^ This includes also 1 case of family and allowance in Calabria, and 1 case of family and “society” in Marche region; ^2^ 1 case of family and private service, 1 society, 1 volunteering, 3 “do not know”.

**Table 5:** Future housing solutions ^1^ and gender, living situation, mobility and supports received (*n*; %).

Sample Characteristics	Home		Home with PCA		Nursing Home		Total	
	*n*	%	*n*	%	*n*	%	*n*	%
**Gender**								
Male	16	53	7	23	9	30	30	100
Female	45	50	17	19	27	30	90	100
**Living Situation**								
Alone	45	48	17	18	31	33	93	100
With PCA	16	59	7	26	5	19	27	100
**Mobility**								
Only in the home	24	50	10	21	14	29	48	100
Additionally, outside the home with help	37	51	14	19	22	31	72	100
**Level of physical limitations ** ^**2**^								
Mild	14	47	9	30	10	33	30	100
Moderate	17	52	6	18	9	27	33	100
High	15	56	1	4	9	33	27	100
Very high	15	50	8	27	8	27	30	100
**Supports ** ^**3**^								
Family	48	51	22	23	30	32	94	100
Public service	21	49	4	9	14	33	43	100
Total respondents ^4^	61	51	24	20	36	30	120	100

^1^ Only three main solutions are reported in the table; ^2^ Mild = no activities “not able”, Moderate = one–two, High = three–four, Very high = five or more; ^3^ Support from both family and public service is possible; ^4^ More future hosing solutions are referred by respondents.

**Table 6:** Caring for older people: family or public responsibility ^1^, by gender, living situation, mobility and supports received (*n*; %).

Sample Characteristics	Family		Family/Public Service		Public Service		Total	
	*n*	%	*n*	%	*n*	%	*n*	%
**Gender**								
Male	12	40	6	20	10	33	30	100
Female	38	42	32	36	16	18	90	100
**Living Situation**								
Alone	39	42	28	30	21	23	93	100
With PCA	11	41	10	37	5	19	27	100
**Mobility**								
Only in the home	19	40	16	33	10	21	48	100
Additionally, outside the home with help	31	43	22	31	16	22	72	100
**Level of physical limitations ** ^**2**^								
Mild	15	50	8	27	7	23	30	100
Moderate	14	42	12	36	6	18	33	100
High	10	37	11	41	5	19	27	100
Very high	11	37	7	23	8	27	30	100
**Supports ** ^**3**^								
Family	38	40	33	35	18	19	94	100
Public service	14	33	15	35	10	23	43	100
Total respondents ^4^	50	42	38	32	26	22	120	100

^1^ “Other” option (6 cases) is not included in the table.^2^ Mild = no activities “not able”, Moderate = one–two, High = three–four, Very high = five or more; ^3^ Support from both family and public service is possible; ^4^ Only one option per respondent is recorded.

**Table 7:** Future housing solutions and family/public responsibility of caring for older people (*n*; %).

Future Housing Solutions ^2^	Responsibility of Caring for Older People ^1^							
	Family		Family/Public Service		Public Service		Total	
	*n*	%	*n*	%	*n*	%	*n*	%
Home	32	52	19	31	7	11	61	100
Home with PCA	8	33	6	25	10	42	24	100
Nursing home	12	33	12	33	9	25	36	100
Total respondents	50	42	38	32	26	22	120	100

^1^ “Other” option (6 cases) is not included in the table, and only one option per respondent is recorded; ^2^ Only three main housing solutions are reported in the table, and more options are referred by respondents.

## Data Availability

The quantitative data presented in this study are openly available in Mendeley at https://doi.org/10.17632/3n83mdt4cd.1 (accessed on 10 June 2022). The thematic charts with the original verbatim transcriptions are not publicly available due to privacy/ethical restrictions, that is to their containing information that could compromise the privacy/anonymity of research participants. (e.g., include names of persons and locations and other potential identifiers of respondents).
